# Thromboinflammation as bioactivity assessment of H_2_O_2_-alkali modified titanium surfaces

**DOI:** 10.1007/s10856-019-6248-4

**Published:** 2019-05-24

**Authors:** Gry Hulsart-Billström, Oscar Janson, Håkan Engqvist, Ken Welch, Jaan Hong

**Affiliations:** 1 Department of Engineering Sciences, Division of Applied Material Science, Uppsala University, 751 21 Uppsala, Sweden; 2 Department of Engineering Sciences, Division of Nanotechnology and Functional Materials, Uppsala University, 751 21 Uppsala, Sweden; 3 Department of Immunology, Genetics and Pathology, Rudbeck Laboratory C5, Uppsala University, 75185 Uppsala, Sweden

## Abstract

The release of growth factors from platelets, mediated by the coagulation and the complement system, plays an important role in the bone formation around implants. This study aimed at exploring the thromboinflammatory response of H_2_O_2_-alkali soaked commercially pure titanium grade 2 discs exposed to whole human blood, as a way to assess the bioactivity of the discs. Commercially pure titanium grade 2 discs were modified by soaking in H_2_O_2_, NaOH and Ca(OH)_2_. The platelet aggregation, coagulation activation and complement activation was assessed by exposing the discs to fresh whole blood from human donors. The platelet aggregation was examined by a cell counter and the coagulation and complement activation were assessed by ELISA-measurements of the concentration of thrombin-antithrombin complex, C3a and terminal complement complex. The modified surface showed a statistically significant increased platelet aggregation, coagulation activation and complement activation compared to unexposed blood. The surface also showed a statistically significant increase of coagulation activation compared to PVC. The results of this study showed that the H_2_O_2_-alkali soaked surfaces induced a thromboinflammatory response that indicates that the surfaces are bioactive.

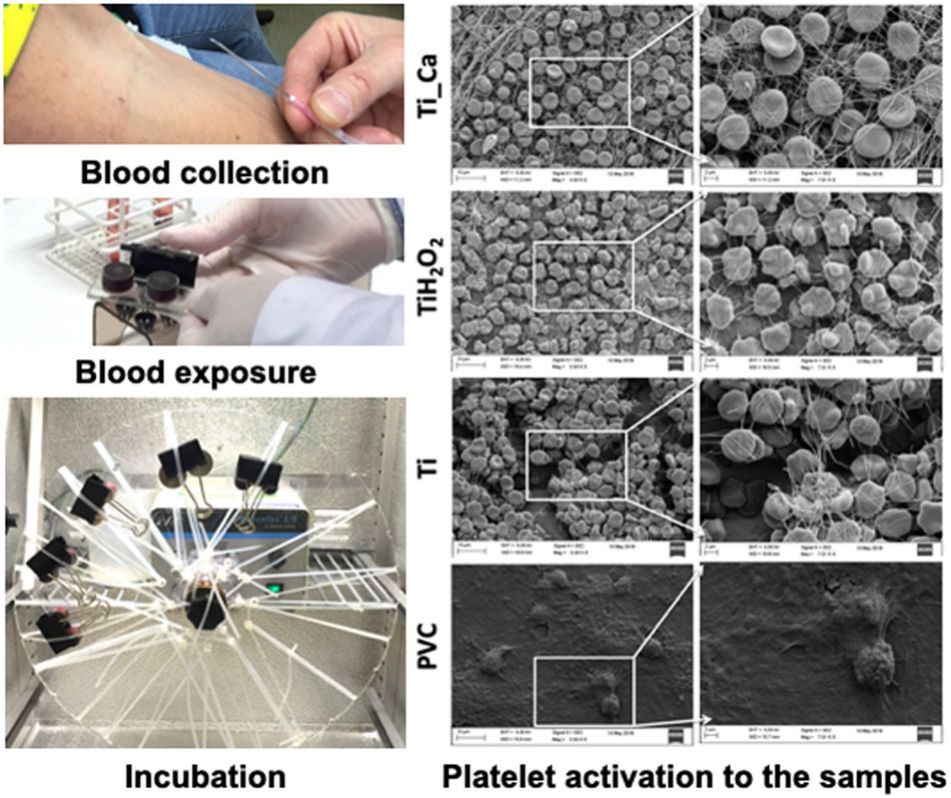

## Introduction

Bacterial infections in conjunction with dental and orthopedic devices are a big concern in healthcare. The material surface that is implanted in the body constitutes an attractive site for bacterial biofilm formation, which can lead to very intractable chronic infections. Consequently, antibiotic treatments are normally used as prevention, both locally and systemically. However, antibiotic treatments often induce adverse side effects and some bacterial strains have been seen to develop resistance against multiple antibiotics, e.g., methicillin-resistant *Staphylococcus aureus* [1]. Therefore, there exists a tremendous incentive to develop new antibacterial treatments for biomedical purposes.

Titanium is a material that has been employed in biomedical implants since the middle of the last century. An advantage of titanium over other metals for use in dental and orthopedic applications is its ability to Osseo integrate, i.e., to form a direct structural and functional connection to the bone. This can be considered to be a specific case of bioactivity, which is the integration of the foreign, synthetic material through interaction with living tissue. Different coatings, chemical treatments, surface roughness and machining techniques can alter the bioactivity of the titanium implants [2]. Titanium has a thin native passivating oxide layer of roughly 5 nm that protects the titanium surface from corrosion. The thickness of the layer can be increased by different methods, e.g., via anodization, heat treatment or chemical surface modification. One such surface modification is hydrogen peroxide soaking of the titanium surface at 80 °C for 1 h, which forms an amorphous titanium peroxy gel layer that can release H_2_O_2_ when degraded and can produce a bactericidal or bacteriostatic effect [3, 4], but this surface is not bioactive and a fibrous layer between the implant and bone is likely to form if implanted in bone. If the implant surface has, for example, an antibacterial coating that can impair the bioactivity/osseointegration, it becomes necessary to increase the bioactivity if the surface is to be used for biomedical applications. Increasing the bioactivity can be done by changing the micro and nano roughness, or by forming a negatively charged, superhydrophilic surface by, for example, soaking in an alkali solution [5]. Sterilisation by autoclaving has been shown to increase the in vitro bioactivity [6].

The in vitro bioactivity can be assessed by soaking the surface in simulated body fluid (SBF) or phosphate buffered saline (PBS) with similar mineral content to blood [7]. The degree of hydroxyapatite layer formation can subsequently be examined by scanning electron microscopy (SEM) [8, 9]. To test the bioactivity in vivo, the most common model used is the rabbit model. Results from animal testing are, however, not always 100% transferable to human conditions [10], and for ethical reasons it is desirable to minimize the amount of animal testing. Several new replacement models are being developed and one model that has been thoroughly investigated over the last decades is the whole blood assay. Here the initial immune response is monitored in terms of coagulation and complement activation. This initial response has a paramount impact on the following osseointegration. For example, in a study by Thor et al., bone formation was shown when dental implants were placed under sinus mucosal lining, at the surface where blood clots formed [11].

The intrinsic pathway of coagulation is the main engager of platelets and is activated by factor XII, which through several activation steps forms thrombin and fibrin. Platelets attach to fibrin and release the growth factors β-thromboglobulin (β-TG), platelet derived growth factors (PDGF) and transforming growth factor-β (TGF-β). These growth factors released from platelets mediate bone formation and thus determining the platelet activation on an implant surface is an excellent indirect method of assessing the bioactivity of the implant. In parallel, the complement system is activated by an implant surface initially by the classical pathway and then continued through the alternative pathway [12]. The classical pathway is initiated when C1 binds to IgG at the material surface and subsequently C3 and C5 are cleaved and result in the terminal complement complex (TCC). Conformational change of C3 occurs in contact with surfaces resulting in amplification of the complement cascade. In absence of a pathogenic membrane it forms sC5b-9. The complement system can also be activated by platelets [13].

It has been shown that titanium in contact with whole blood is more thrombogenic than, for example, steel, polyvinyl chloride (PVC), aluminium and zirconium [14]. The aim of the present study was to examine the thromboinflammation as platelet activation, mediated by the intrinsic coagulation- and complement system on H_2_O_2_ and alkali modified commercially pure titanium grade 2 surfaces. This is an indirect way to assess the bioactivity on the surface of these modified titanium discs. This relationship was examined using the replacement model developed by Hong et al. [15], employing the whole blood response of human blood when exposed to surfaces.

## Materials and methods

### Materials

Commercially pure titanium grade 2 discs with a diameter of 16 mm and thickness of 0.5 mm (Optimel Elektronik och Plåtteknik AB, Uppsala, Sweden) were first ground with 1200 grit SiC paper, then polished with a 6 µm diamond slurry. The discs were subsequently sonicated in a serial sequence of acetone, 96% ethanol and Milli-Q H_2_O for 15 min each. Two different test groups were produced for this study. Both groups were soaked in 30% v/v H_2_O_2_ for 1 h at 80 °C. One group did not receive further treatment (denoted Ti_H_2_O_2_) while the other group was subsequently sequentially soaked in 5 M NaOH and 0.1 M Ca(OH)_2_ for 15 min each at room temperature (denoted Ti_Ca). Positive controls consisted of commercially pure titanium grade 2 discs (denoted Ti) and negative controls consisted of polyvinylchloride discs (denoted PVC).

### Surface roughness

Before and after the surface treatment, the surface roughness was assessed with a Dektak XT Advance (Bruker, Tucson, AZ, USA) profilometer. Each disc was assessed along six lines. Ten discs were measured for the two test groups (*n* = 10), and three for both control groups (*n* = 3).

### Contact angle

Hydrophilicity of the disc surfaces was assessed by measuring the contact angle of a drop (approximately 2 µl) of Milli-Q water placed on the surface. A digital image of the drop from the side was taken and the angle between the liquid-surface interface and liquid-vapor interface where the liquid-vapor interface meets the disc surface was measured. The contact angle on both sides of the drop were averaged and taken as the measured value. Two discs from each test and control group were tested and two drops were applied to each disc (*n* = 4 for each group).

### Heparinisation

The slide chamber, tubes and tips used in the replacement model developed by Hong et al. [15] were coated with the Corline heparin surface (Corline Systems AB, Uppsala, Sweden) according to the manufacturer’s recommendation [16]. The surface was first incubated with a polymeric amine compound (PAV, Corline Systems AB, Uppsala, Sweden) before adding a heparin conjugate that becomes irreversibly bound by multiple ionic interactions. This procedure was repeated once, resulting in a double-layered heparin coating with a heparin surface concentration of 0.5 g/cm^2^ and giving a binding capacity of 2-4 pmol/cm^2^ antithrombin described earlier by Gong et al. [16].

### Blood sampling

Roughly 20 ml blood was obtained from five healthy individuals each. The blood was obtained in a heparinized open system and collected in falcon tubes containing heparin, having a final concentration of 0.5 IU heparin/ml. Ethical approval for the blood test was obtained from the regional ethic committee (reference number 2008/264). Informed consent was given from the blood donors before the experiment.

### In vitro whole blood model

An in vitro whole blood model was used to investigate the interaction between the blood and sample discs. Before testing, test discs were put into sterile pouches and autoclaved at 125 °C for 1 h while the positive (titanium) and negative (PVC) discs were cleaned in 5% (w/v) ammonium persulphate for 60 min at 60 °C. This in vitro whole blood model is described in detail elsewhere [15], but in brief, 1.4 ml blood was transferred to heparinized poly(methyl methacrylate) (PMMA) slide chambers containing two wells. The sample discs were placed on top of the chamber and fixed with a paper clip. As a 0 min sample and reference point, 1 ml of blood from each donor was introduced into Eppendorf tubes containing ethylenediaminetetraacetic acid (EDTA) at a concentration of 4 mM, (referred to as initial). The blood-containing slide chambers with discs were incubated in 37 °C for 60 min under rotation on a wheel at 22 revolutions/min.

### Platelet activation

After incubation, the blood was transferred to Eppendorf tubes containing 4 mM EDTA, and the samples were analyzed for platelet numbers in a XP-300 Hematology Analyzer (Sysmex Corporation, Japan). The remaining blood was circulated in 4500 g for 15 min at 4 °C, after which the plasma was collected and stored in −70 °C for further analysis. All samples were measured in duplicates and blood from five donors was used.

### Scanning electron microscopy (SEM)

After the incubation the samples were fixated in 2.5% (v/v) glutaraldehyde. After 1 h fixation the samples were rinsed three times in phosphate buffered saline (PBS) and then immersed for 10 min each in ethanol solutions at concentrations of 30, 50, 70 and 96% (v/v). Afterwards the discs were immersed in hexametyldisilane (HMDS) for 15 min and left to dry for a couple of hours. The discs were then sputter-coated with an Au/Pd layer for increased signal and reduced charging effect. The disc surfaces were finally imaged at comparable places on each sample in a LEO 1530 or 1550 scanning electron microscope (Zeiss, Oberkochen, Germany) using the secondary electron detector and operated at 5 or 10 kV.

### Enzyme-linked immunosorbent assay (ELISA)

Enzyme-linked immunosorbent assay (ELISA) was employed to evaluate the complement and coagulation activation markers of thrombin-antithrombin complexes (TAT), C3a fragment, and terminal complement complex (TCC). All three ELISAs used PBS containing 0.1% (v/v) of Tween 20. The samples were diluted in PBS containing 1% (w/w) bovine serum albumin, 0.1% (v/v) Tween 20 and 10 mM EDTA. 3,3′,5,5′-tetramethyl-benzidine was used as a substrate for all ELISAs. The absorbance was measured at the wavelength of 450 nm using a micro-plate reader (Tecan Group Ltd, Switzerland).

#### Thrombin-antithrombin complexes (TAT)

Thrombin-antithrombin complexes plasma levels were analyzed by sandwich-ELISA. The complexes were bound in wells coated with anti-human thrombin antibody (Enzyme Research Laboratories Inc., USA) that were diluted 1/20. Human serum diluted in normal EDTA plasma was used as a standard. The bound TAT was detected with horseradish peroxidase (Enzyme Research Labs Inc., USA).

#### C3a

C3a levels were analyzed in plasma by the method described by Nilsson Ekdahl et al. [17]. The monoclonal antibody 4SD17.3 was used as the coating antibody and biotinylated anti-human C3a as detection, followed by HRP-conjugated streptavidin for signal. Zymosan-activated serum, calibrated against a solution of purified C3a, was used for the standard curve. The control contained zymosan-activated serum with a 1/500 dilution. Anti-C3a monoclonal antibody 4SD17.3 (in house) was used as capture antibody. Bound C3a in plasma samples was detected with biotinylated anti-C3a antibody (in house) followed by HRP-conjugated streptavidin (GE Health- care, Sweden). Zymosan-activated serum, calibrated against a solution of purified C3a, was used as standard.

#### Terminal complement complex (TCC)

Anti-neoC9 monoclonal antibody aE11 (Diatec Monoclonals AS, Norway) was used as capture antibody. TCC in plasma samples was detected by a biotinylated polyclonal anti-C5 antibody (Nordic BioSite AB, Sweden), followed by HRP-conjugated streptavidin (GE Healthcare, Sweden). Zymosan-activated serum was used as standard.

### Statistical analysis

Data were analyzed using the GraphPad Prism software package (version 5.0 f). Ten samples were prepared for the test samples. Two pairs of samples were used for each donor and the mean from each donor was used for statistical analysis (*n* = 5). Values are given as mean ± SD. ANOVA with Tukey’s multiple comparison test were used to determine statistical significance. Values at *p* < 0.05 were considered statistically different.

## Results

### Titanium surface modification

**Fig. 1:**
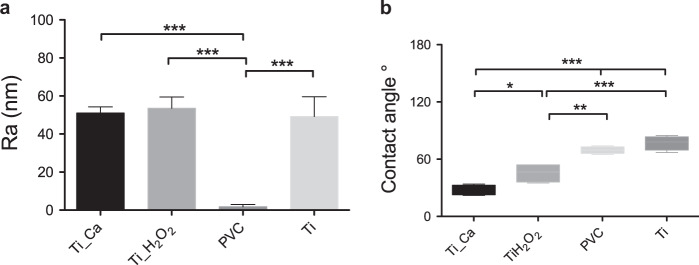
Surface roughness (nm) and contact angle (°) of the different surfaces tested. **a** Surface roughness Ra as mean ± SD (*n* = 10 for test groups and *n* = 3 for control groups, ***p < 0.0001; ANOVA with Tukey’s multiple comparison test). **b** Contact angles as means ± SD (*n* = 4, *p < 0.05, **p < 0.01, ***p < 0.0001; ANOVA with Tukey’s multiple comparison test)

Figure 1 presents results from the surface roughness and contact angle measurements. There was no significant difference in surface roughness between Ti_Ca, Ti_H_2_O_2_ and Ti (*p* < 0.05; ANOVA with Tukey’s multiple comparison test, two-tailed; *n* = 10). The H_2_O_2_-alkali modification of the titanium did not seem to have an effect on the surface roughness. The surface roughness was comparable between the Ti_Ca (51 ± 3), Ti_H_2_O_2_ (54 ± 6) and Ti (49 ± 10). The negative control PVC was significantly smoother (2 ± 2) compared to the titanium groups (***p < 0.0001; ANOVA with Tukey’s multiple comparison test, two-tailed; *n* = 10). The contact angle of the test group surfaces had a significantly lower contact angle compared to the control discs. Ti_Ca had a significantly lower contact angle than Ti_H_2_O_2_.

### Blood testing

#### Imaging

**Fig. 2:**
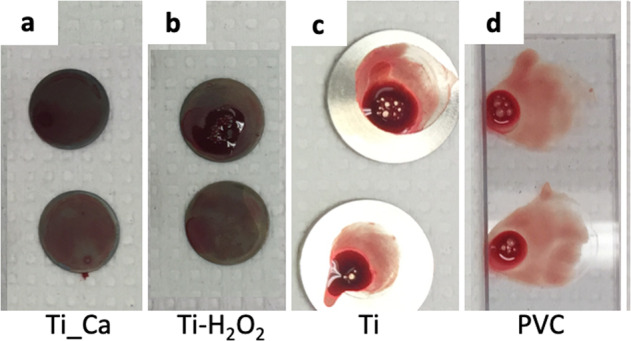
Macroscopic images of the surface activation of the disc surfaces after exposure to whole blood for 60 min at 37 °C. **a** Ti_Ca, **b** Ti_H_2_O_2_, **c** Ti and **d** PVC. The three titanium groups displayed comparable activation and adherence of blood cells and platelets. The negative control of PVC demonstrated low adhesion. Activation and adherence were macroscopically defined as the blood that had adhere onto the surface. The dark aspects on the test groups are due to the heat treatment in H_2_O_2_ at 80 °C

**Fig. 3:**
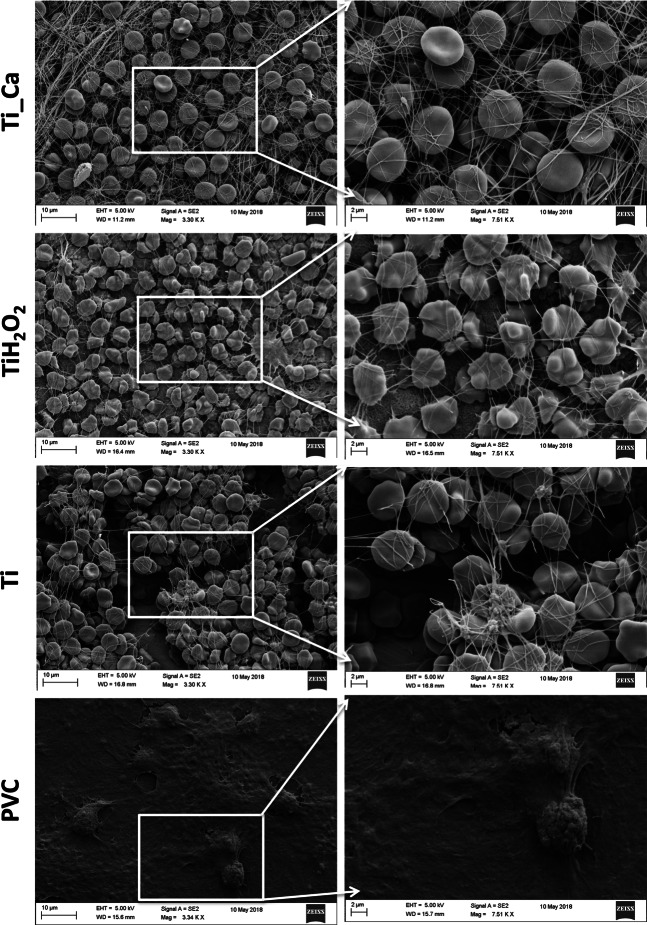
Scanning electron micrographs of disc surfaces upon contact activation. Blood (1.4 ml) containing 0.5 IU heparin/ml was incubated for 60 min in a slide chamber where it was in contact with a Ti_Ca, Ti_H_2_O_2_ Ti-control or PVC disc surface. Right panels show higher magnification of the defined box drawn in the left panels. Both test groups and the positive Ti control displayed comparable activation and adherence of blood cells and platelets while the negative control PVC demonstrated low adhesion

Macroscopic images of the surface activation of the two test groups and positive Ti control after exposure displayed comparable activation and adherence of blood cells and platelets, see Fig. 2. On the other hand, the negative control PVC demonstrated low adhesion. Activation and adherence were defined as the blood adhered to the surface. The dark aspects on the test group were due to the heat treatment in H_2_O_2_ at 80 °C. These results were further confirmed with high magnification using SEM, see Fig. 3. A high amount of blood cells and platelets were bound to the surface of Ti_Ca and Ti_H_2_O_2_ discs, similar to that observed on the positive Ti control. PVC demonstrated low adhesion with only a few cells attaching to the surface.

#### Platelet activation

**Fig. 4:**
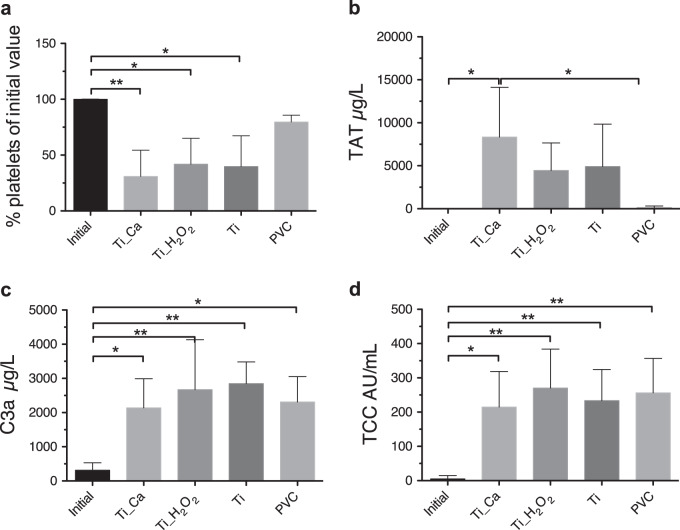
Coagulation activation upon contact with disc surfaces. **a** Percentage of remaining platelets of the initial value prior to exposure. Both test groups and the positive Ti control showed significantly higher platelet activation compared to the initial value (*p < 0.05, **p < 0.01; ANOVA with Tukey’s Multiple Comparison Test). **b** Thrombin-antithrombin complex, Ti_Ca showed significantly higher TAT complexes compared to initial amount and PVC control (*p < 0.05; ANOVA with Tukey’s Multiple Comparison Test). Both C3 generation (**c**) and terminal complement complex were in significantly higher concentrations with all test materials including PVC control when compared to the initial amount (*p < 0.05, **p < 0.01; ANOVA with Tukey’s multiple comparison test). Results are an average value from two wells/donor represented as mean ± SD (*n* = 5)

The platelet activation was measured by the percentage of platelets remaining after 1 h of exposure to the surfaces at 37°. Samples were taken prior to exposure and the initial concentration of platelets was measured. Both modified titanium groups and the Ti control showed significantly higher platelet activation compared to the initial amount (*p < 0.05, **p < 0.01; ANOVA with Tukey’s multiple comparison test, two-tailed; *n* = 5 donors; Ti_Ca = 31% ± 23; Ti_H_2_O_2_ = 42% ± 23; Ti_control_ = 40% ± 27). PVC did not show a significant decrease in platelets compared to the initial value (PVC = 80 % ± 6) (Fig. 4a).

### Enzyme-linked immunosorbent assay (ELISA) of TAT, C3a and TCC

Thrombin–antithrombin (TAT) complex generation was activated by the titanium groups. Ti_Ca showed significantly higher TAT complexes compared to initial amount and the PVC control (*p < 0.05; ANOVA with Tukey’s multiple comparison test) (Fig. 4b). When compared to initial values, complement activation of C3a and TCC was activated on all samples including controls, and both C3 generation (Fig. 4c) and TCC (Fig. 4d) were significantly increased on all materials including PVC control (*p < 0.05, **p < 0.01; ANOVA with Tukey’s multiple comparison test).

## Discussion

In the present study, we have investigated the influence of two surface treatments of titanium on the thrombin generation and subsequent platelet activation in whole blood. We were able to show pronounced activation of blood coagulation with the different titanium surfaces.

The H_2_O_2_ and alkali modified titanium surfaces significantly increased platelet activation mediated by the upregulation of the intrinsic coagulation and complement system. These results prove the modifications to be bioactive in terms of thromboinflammation. Hong et al. showed two decades ago that titanium surfaces induce macroscopic clotting; this was not evident to the same extent when whole blood was exposed to steel or PVC. Similar to our results, thrombin levels increased significantly only in contact with titanium [18]. Thrombin has profound effect on several steps of blood coagulation. The thrombin cleavage of factor XIII to active FXIIIa is important for fibrin polymerization [19]. In addition, it is shown that the active form of FXIII is involved in wound healing and osteoblast matrix secretion and deposition [20, 21]. Nakamura et al. showed in a study that a calcium-releasing surface increased the conversion of FXIII. In our setup we observed significant thrombin formation and strongly reduced platelet count with Ti-Ca.

The effects of thrombin modulate the progression of wound healing by induction of M2a macrophages [22]. It has been shown by Trindade et al. that titanium implants activate the immune system more towards type 2 inflammation, which is associated with M2 macrophages and TH2 T-cells that both are guiding the immune system towards healing [23]. They also found a decrease in bone resorption adjacent to the titanium surfaces. The authors suggest that the titanium is recognized as a foreign body by the immune system and that in reality the bone forming environment is an endogenous attempt to isolate the foreign body from the bone marrow [24].

Hong et al. also discovered pronounced platelet activation with elevated levels of thromboglobulin and PDGF, proteins that are known to promote osteogenesis [18]. In our study the number of free platelets in the blood were reduced by entrapment in the blood clot on all titanium surfaces. Activation of platelets promotes an anti-inflammatory environment by inducing IL-10 production and inhibiting TNF-alpha production by monocytes [25]. Taken together this suggests that platelet activation on titanium surfaces elicit non-inflammatory and osseointegrative properties.

The surface roughness heavily affects the thrombogenicity, with a rougher surface leading to more blood clotting [26]. Therefore it was important to have a smooth surface to be able to examine the effect from the chemical surface modification. The SEM images in Fig. 3 display an ample fibrin formation and adhesion of erythrocytes on the test groups and the Ti disc. This showed that the coagulation cascade had produced high amounts of fibrin. This clearly indicates that both Ti_Ca and Ti_H_2_O_2_ elicit a coagulation response and a thrombogenic response. This is in line with the results of Takemoto et al. that showed that H_2_O_2_ oxidized amorphous titanium dioxide surfaces lead to an increased number of adhesive platelets compared to non-treated and crystalline TiO_2_ surfaces [3, 27].

Additionally, Thor et al. evaluated the thrombogenic response of whole blood in contact with modified titanium surfaces that were either machined, grit-blasted, or fluoride-modified and grit-blasted. They used the same whole blood exposure-setup of 60 min rotation at 22 rpm at 37 °C and proved that blood in contact with Ti alloys resulted in the binding of platelets and highly amplified TAT levels. In addition the fluoride-modified surface had an enhancing effect on the thrombogenic properties of the titanium [28]. The same group concluded that hydrophilic modification of titanium surfaces enhance the thrombogenic properties and thus promote bone integration of titanium implants [26]. Ikada and Takemoto et al. showed that the platelet adhesion increased with higher contact angle and reached a maximum at 70–80°, and then declined with higher contact angles [27, 29].

The volume of blood inserted into the wells was chosen to be 1.4 ml to a total volume of the well of 1.6 ml. This gave rise to an air bubble, which by enhanced stirring, increased the conformational change of C3 leading to activation of the alternative pathway. Both C3 generation and TCC were significantly increased on all test materials including PVC control when compared to the initial value, which seems vital for successful fracture healing. Recently it has been shown that the complement is crucial in bone development and complement receptors are expressed both by immune cells, osteoblasts and chondroblasts [30]. Ehrnthaller et al. demonstrated that the final step in complement is crucial for fracture healing. They used either C3 or C5 deficient mice to investigate healing after osteotomy in the absence of C3 and C5. The healing was delayed but still successful in the C3 deficient mice in contrast to the C5 deficient mice, which displayed incomplete bone healing. They proved that the C5a was activated in C3 deficient mice [31].

## Conclusions

To summarize, both the modified and non-modified titanium surfaces showed extensive coagulation indicating bioactivity of the material. The ability to keep comparable thrombogenic effects as unmodified titanium suggests that the surface modifications are promising candidates for further in vivo testing as bioactive implant surfaces.
